# Liver Function Test Abnormalities in Experimental and Clinical *Plasmodium vivax* Infection

**DOI:** 10.4269/ajtmh.20-0491

**Published:** 2020-08-17

**Authors:** Anand Odedra, Lachlan Webb, Louise Marquart, Laurence J. Britton, Stephan Chalon, Joerg J. Moehrle, Nicholas M. Anstey, Timothy William, Matthew J. Grigg, David G. Lalloo, Bridget E. Barber, James S. McCarthy

**Affiliations:** 1 QIMR Berghofer Medical Research Institute, Brisbane, Australia;; 2 Liverpool School of Tropical Medicine, Liverpool, United Kingdom;; 3 Department of Gastroenterology and Hepatology, Princess Alexandra Hospital, Brisbane, Australia;; 4 School of Medicine, The University of Queensland, Brisbane, Australia;; 5 Medicines for Malaria Venture, Geneva, Switzerland;; 6 Global and Tropical Health Division, Menzies School of Health Research, Darwin, Australia;; 7 Clinical Research Centre, Queen Elizabeth Hospital, Kota Kinabalu, Malaysia;; 8 Gleneagles Hospital, Kota Kinabalu, Malaysia

## Abstract

Liver transaminase elevations after treatment in malaria volunteer infection studies (VISs) have raised safety concerns. We investigated transaminase elevations from two human *Plasmodium vivax* VISs where subjects were treated with chloroquine (*n* = 24) or artefenomel (*n* = 8) and compared them with studies in Thailand (*n* = 41) and Malaysia (*n* = 76). In the VISs, alanine transaminase (ALT) increased to ≥ 2.5 × upper limit of normal (ULN) in 11/32 (34%) volunteers, peaking 5–8 days post-treatment. Transaminase elevations were asymptomatic, were not associated with elevated bilirubin, and resolved by day 42. The risk of an ALT ≥ 2.5 × ULN increased more than 4-fold (odds ratio [OR] 4.28; 95% CI: 1.26–14.59; *P* = 0.02) for every log_10_ increase in the parasite clearance burden (PCB), defined as the log-fold reduction in parasitemia 24 hours post-treatment. Although an elevated ALT ≥ 2.5 × ULN was more common after artefenomel than after chloroquine (5/8 [63%] versus 6/24 [25%]; OR 5.0; 95% CI: 0.91–27.47; *P* = 0.06), this risk disappeared when corrected for PCB. Peak ALT also correlated with peak C-reactive protein (*R* = 0.44; *P* = 0.012). Elevations in ALT (≥ 2.5 × ULN) were less common in malaria-endemic settings, occurring in 1/41 (2.5%) Thai patients treated with artefenomel, and in none of 76 Malaysians treated with chloroquine or artemisinin combination therapy. Post-treatment transaminase elevations are common in experimental *P. vivax* infection but do not appear to impact on participant safety. Although the mechanism of these changes remains uncertain, host inflammatory response to parasite clearance may be contributory.

## INTRODUCTION

Liver enzyme elevations have been recently reported in both *Plasmodium falciparum*^1^ and *Plasmodium vivax*^2^ malaria volunteer infection studies (VISs), otherwise known as controlled human malaria infection studies. These studies entail intentional infection of healthy volunteers with *Plasmodium* parasites, by mosquito bites,^3^ direct injection of sporozoites,^4^ or by intravenous inoculation of parasitized red blood cells.^2,5^ The latter bypasses the liver stage of infection and is referred to as the induced blood-stage malaria (IBSM) model. VISs are increasingly being used to evaluate antimalarial vaccine and drug candidates^4,6^ and have been established for *P. falciparum*,^7^ *P. vivax*,^2,8^ and *Plasmodium malariae*.^5^ However, recent reports of elevated liver enzymes post-treatment have raised concerns related to the safety of healthy volunteers and have the potential to impede antimalarial drug development because of difficulties distinguishing drug-induced liver injury (DILI) from the effect of malaria.

DILI is the most common cause of cessation of development of a new chemical entity (NCE) for safety reasons.^9^ DILI can be divided into intrinsic injury, which is related to a direct drug effect and is typically dose dependent, and idiosyncratic injury, which occurs in specific susceptible individuals. The latter is typically not dose related and is, therefore, more difficult to predict. Most drugs that cause severe DILI do so infrequently, typically requiring at least a few thousand exposed subjects to demonstrate the effect.^9^ Therefore, close attention must be paid to lower degrees of DILI during preclinical and clinical drug development. Hence, liver function test (LFT) abnormalities must be carefully evaluated during malaria VISs where an NCE is under development. Incorrect attribution of liver injury to an NCE can lead to cessation of further development; conversely, attributing the liver injury solely to malaria may risk failure to detect DILI. Currently, there is no reliable biomarker to distinguish DILI from other causes of liver injury. Therefore, better understanding of the factors associated with post-treatment LFT abnormalities in malaria VISs is required.

Malaria hepatopathy is a common phenomenon in natural malaria infection, occurring in both *P. falciparum* and *P. vivax* infections*.* It is defined as a bilirubin > 2.5 × upper limit of normal (ULN) and an increase in transaminase > 3 **×** ULN. These LFT elevations generally peak around the time of diagnosis and before commencement of antimalarial chemotherapy.^10–14^ By contrast, a distinct pattern of LFT abnormality has been reported in *P. falciparum*^1^ and *P. vivax* VISs.^2^ This is characterized by transaminase elevation without an increase in bilirubin, with a peak observed approximately 2–6 days post-treatment.^15–17^ Similar changes have been described in natural *P. falciparum*^18,19^and *P. vivax* infections,^18^ although they have been reported less commonly than malaria hepatopathy. The differences between the malaria hepatopathy and post-treatment LFT changes suggest that they may be mediated by different mechanisms. The mechanisms underlying post-treatment liver injury are incompletely understood. Possible contributing factors include an increased inflammatory response following malaria treatment, release of hepatotoxic iron products from red cells, oxidative damage, and, potentially, a hepatotoxic effect of antimalarial drugs or other concomitant drug treatment (including acetaminophen).^15^

The aim of our study was to describe in detail the LFT abnormalities observed in two *P. vivax* IBSM studies and to evaluate possible risk factors. One of these studies entailed treatment with the investigational antimalarial artefenomel, and the other with the licensed drug chloroquine. Neither artefenomel nor chloroquine has been associated with hepatotoxicity when used as monotherapy in standard dose regimens in healthy adults^20–23^ or in children with malaria.^24^ However, post-treatment LFT abnormalities have been reported in malaria patients treated with artefenomel in malaria-endemic settings.^25,26^ We compared findings from the *P. vivax* VISs with data from a published phase IIA open-label study of artefenomel treatment of malaria in Thailand^25^ and from studies of chloroquine or artemisinin combination therapy conducted in Malaysia.^27,28^

## MATERIALS AND METHODS

### Subjects and study design.

Data for the *P. vivax* IBSM analysis were obtained from 32 malaria-naive subjects who participated in two clinical trials at the Queensland Institute of Medical Research (QIMR) Berghofer between March 2016 and April 2017. The results of one of these studies, which used chloroquine^29^ and artefenomel^30^ as antimalarial treatment have been published. An article describing the results of the other study (NCT02573857), which used the experimental compound artefenomel as antimalarial treatment, is currently under review. Key details are included in Table 1. Subjects were all healthy adults aged between 18 and 55 years. Subjects with a history or clinical signs of liver disease were excluded. All subjects had alanine transaminase (ALT), aspartate transaminase (AST), and total bilirubin levels within the normal range at baseline. Subjects were inoculated intravenously with the *P. vivax* isolate HMP-013.^29^ In the artefenomel study, eight subjects were treated with a single 200 mg dose of the drug 10 days post-inoculation.^30^ Data from previous studies had indicated that a 200 mg dose would be subcurative,^31^ and as expected, 7/8 subjects had recrudescent parasitemia; they subsequently received a standard course of artemether/lumefantrine on day 21–28. In the chloroquine study, 24 subjects were treated with a standard 3-day course of chloroquine, totaling 25 mg/kg, beginning on day 8 (*n* = 8 subjects), day 9 (*n* = 1 subject), or day 10 (*n* = 15 subjects). All subjects in the chloroquine study cleared parasites.

**Table 1:** Induced blood-stage malaria clinical trials details

Drug	Artefenomel	Chloroquine			
Cohort (*n*)	Artefenomel (8)	Chloroquine C1 (8)	Chloroquine C2a (6)†	Chloroquine C2b (2)†	Chloroquine C3 (8)
Inoculation dose size (parasites)	524,000	720,000	722,000	740,000	782,000
Treatment regimen	Single dose 200 mg	3 Days*	3 Days*	3 Days*	3 Days*
Day of treatment post-inoculation	Day 10	Day 8	Day 10 (one subject treated on day 9)	Day 10	Day 10
Acetaminophen and/or ibuprofen use	Allowed both	Allowed both, encouraged ibuprofen only, and only reported ibuprofen use	Ibuprofen	Ibuprofen	Acetaminophen

* Three-day standard oral curative treatment regimen dosed on a weight basis to a total of ∼25 mg/kg.

† Cohorts 2a and 2b were combined into chloroquine cohort C2 for the statistical analysis as the conduct and characteristics of the cohorts were identical apart from the inoculum dose.

Individual patient-level biochemistry results were made available from a previously published phase IIA open-label study.^25^ This study had enrolled patients at the Hospital for Tropical Diseases in Bangkok and Shoklo Malaria Research Unit located on the northwestern border of Thailand between October 2010 and May 2012. Data from 41 adult patients with microscopically confirmed *P. vivax* mono- (*n* = 40) or mixed infection (*n* = 1) were analyzed. LFTs were measured before artefenomel treatment, 4 hours post-treatment, and on days 1, 2, and 7.

In the study undertaken in Malaysia, adults and children > 1 year of age with *P. vivax* monoinfection were enrolled from July 2013 to November 2015 as part of a randomized controlled trial^27^ or a prospective observational study,^28^ both conducted at Kota Marudu, Kudat, and Pitas district hospitals. LFTs were undertaken on samples from 85 patients. All had ALT levels tested on days 0 (before treatment) and 7, whereas AST was measured in 19/85 and 85/85 subjects on day 0 and day 7, respectively. Total bilirubin levels were tested in 76/85 subjects on day 0 and 85/85 subjects on day 7.

### Parasitemia measurements.

Parasitemia was measured before treatment and at multiple time points throughout the VISs by 18S-qPCR^32^ and analyzed on the log_10_ scale. The parasite clearance rate was reported using the parasite clearance half-life (PCt_1/2_) in hours, as previously reported.^33^

For the artefenomel VISs, the PCt_1/2_ was censored at parasitemia nadir, ∼36 hours post-treatment, and before recrudescence. Although chloroquine was administered over 3 days, the initial parasite PCt_1/2_ was assumed to represent a continuous pharmacodynamic effect.^29^

A variable was calculated to capture the biomass of parasites cleared in the first 24 hours after treatment. This variable, termed as the parasite clearance burden (PCB_peak-24_) was calculated by subtracting the parasitemia at 24 hours posttreatment initiation from the peak parasitemia on the log_10_ scale and is reported as a log-fold change. The 24-hour cutoff was selected because most parasites were cleared over this interval, yet parasitemia remained measurable. The peak parasitemia occurred at or around the time of treatment for all subjects.

### Markers of host response.

Temperature, malaria clinical score, and C-reactive protein (CRP) were measured as markers of host response. Temperatures < 37.5°C were considered normal. The malaria clinical score has been developed as a tool for assessing the clinical severity of malaria in IBSM studies^34^ and forms part of the criteria for treatment initiation (Supplemental Material). The maximum temperature and clinical scores were taken from values recorded within 3 days pre- and post-treatment to ensure the changes were due to malaria infection and treatment and not to an alternative cause.

### Laboratory parameters.

LFT and hematology results from the IBSM subjects were taken at screening, pre-inoculation, pre-treatment, 72 hours post-treatment, 5–8 days post-treatment, and at the end of study (EOS). CRP levels were retrospectively measured on a total of 31 stored plasma and 16 stored serum samples in the artefenomel cohort, and 62 stored plasma and serum samples in the chloroquine cohorts. Additional CRP results were available from tests undertaken for clinical assessments. The peak ALT level was assessed as the primary measurement of hepatocyte injury because of its greater liver specificity than AST.^35^ Peak ALT was categorized into ≥ 2.5 × ULN and < 2.5 × ULN (ULN = 40 IU/L for males and 30 IU/L for females) in line with the World Health Organization guidelines.^36^

### Statistical analysis.

Data were analysed using Stata v.15 (StataCorp 2017, College Station, TX),^37^ and figures were produced using GraphPad Prism v.8, San Diego, CA.^38^ Descriptive statistics of frequency and percentage were given for categorical measures, and continuous variables were presented as mean and 95% CI or median and interquartile range (IQR), depending on whether the variable was normally distributed or not. For the VIS participants, maximum and minimum values for all parameters (LFTs, inflammatory, lactate dehydrogenase [LDH], and parasitemia) were identified from inoculation to drug treatment and from inoculation to the EOS. Correlations between continuous variables were assessed by Pearson’s correlation of log_10_-transformed data. Logistic regression analysis was used to test for associations between explanatory parameters and peak ALT ≥ 2.5 × ULN. A fixed cohort effect was added to the regression models to account for inherent differences between cohorts. The Mann–Whitney test was used to assess for difference in ALT values between the two drug treatments.

A backward stepwise regression was used to create two multivariable logistic models for predictors of developing a peak ALT ≥ 2.5 × ULN. Any variable that was significant (*P* < 0.05) in the univariate logistic regression was included in the backward stepwise regression. In the first multivariable model, the parasite clearance rate was forcibly retained into the final model; in the second multivariable model, the PCB was included. For the Thailand and Malaysia datasets, continuous measures were described using mean (95% CI), and paired *t*-tests on the log_10_ scale of the measure were used to test the differences in LFT results between day 0 (pre-treatment for Thailand artefenomel) and day 7.

### Review of subject-level data.

The corresponding author reviewed the source documents of all 32 subjects involved in the *P. vivax* IBSM studies to exclude an alternative diagnosis such as alcohol consumption and to evaluate concomitant medication use such as acetaminophen.

## RESULTS

### Volunteer infection studies.

All eight subjects enrolled in the artefenomel study were male compared with a more even gender ratio in the chloroquine study where 13/24 (54%) of subjects were males. There was no difference in the age of subjects recruited in the artefenomel and chloroquine studies (median [IQR] 23.5 [22–25.8] years and 24.5 [20–30] years, respectively).

#### Liver function test abnormalities.

**Figure 1.**
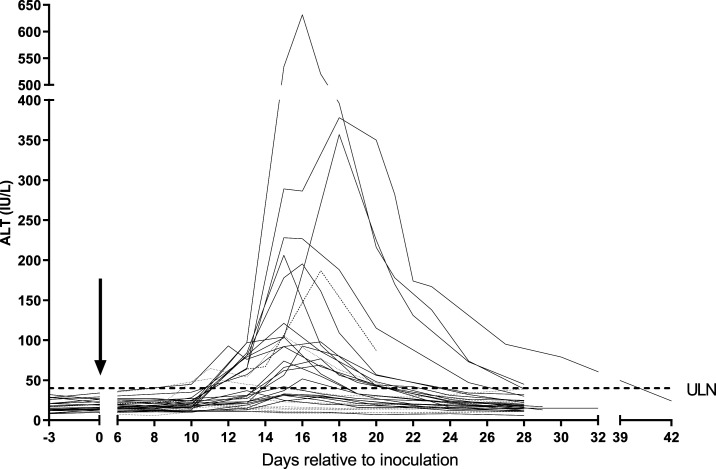
Course of ALT over time in *Plasmodium vivax*–induced blood-stage malaria. Changes in ALT levels over time for all 32 subjects. Each of the 32 subjects are represented by a single trace. Day 0 represents the day of inoculation (arrow). Those subjects represented by dotted lines were from chloroquine cohort C1 (*n* = 8), and all were treated 8 days post-inoculation. The dashed line represents subject C13 (R013) from chloroquine cohort C2 who was the only subject treated on day 9 post–inoculation. The ULN for ALT in male subjects (40 IU/L) has been included for reference (dashed horizontal line). ALT = alanine transaminase; ULN = upper limit of normal.

Following antimalarial treatment, an ALT ≥ 2.5 × ULN occurred in 11/32 (34%) subjects. Alanine transaminase elevations generally exceeded AST (correlation peak ALT to peak AST *R* = 0.99; *P* < 0.001). Levels typically began to increase 3 days after treatment, peaking between days 5 and 8 after treatment and resolving between day 11 and day 32 after treatment (Figure 1). The highest ALT observed was 632 IU/L (15.8 × ULN), occurring 6 days post-treatment. All subjects remained clinically well. There was no relationship between age or gender and LFT elevations. No volunteer had significant elevations in alkaline phosphatase or bilirubin, and Hy’s law was not invoked. Gamma glutamyl transferase was measured in the artefenomel-treated subjects only; all results were within the normal range. Creatine kinase was measured in three artefenomel-treated subjects and one chloroquine-treated subject at or within 3 days of the peak ALT; all were within normal range. Mild elevations in LDH of up to 1.6 × ULN were observed in 19/32 subjects. Elevations in CRP levels were common and typically peaked at 2–3 days post-treatment. Peak ALT occurred before recrudescence of parasitemia in subjects treated with artefenomel.

**Figure 2.**
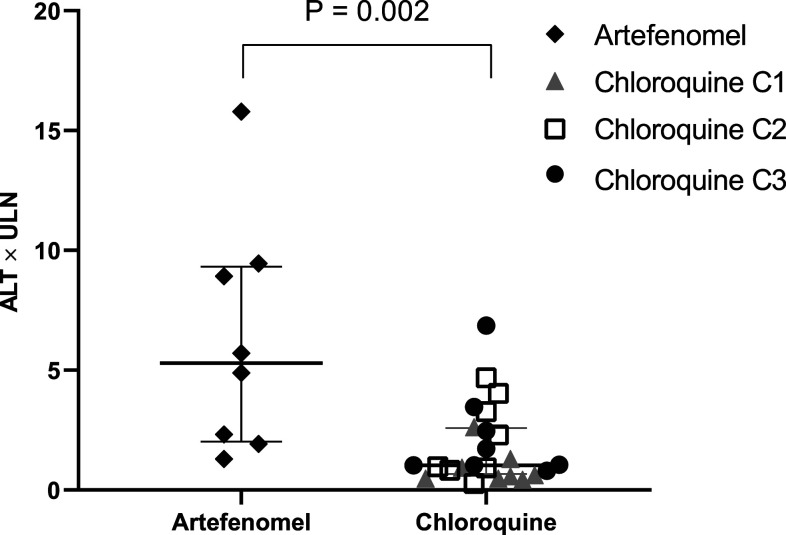
Peak ALT × ULN for each induced blood-stage malaria subject per treatment. Peak ALT **×** ULN with median and interquartile range for subjects treated with artefenomel (*n* = 8) and chloroquine (*n* = 24). Subjects treated with chloroquine came from three cohorts (cohort 1, *n* = 8; cohort 2, *n* = 8; cohort 3, *n* = 8). ALT = alanine transaminase; ULN = upper limit of normal.

The peak ALT was greater among the eight subjects administered with artefenomel (median 5.3 [IQR: 2.2–9.1] × ULN) than that among the 24 subjects who were given chloroquine (1.0 [IQR: 0.8–2.5] × ULN; *P* = 0.002) (Figure 2). Likewise, a greater proportion of subjects in the artefenomel group had a peak ALT of ≥ 2.5 × ULN (5/8 [63%] versus 6/24 [25%]; odds ratio [OR] 5.0; 95% CI: 0.91–27.47; *P* = 0.064).

#### Parasite measures.

Peak parasitemia was associated with peak ALT (*R* = 0.39; *P* = 0.027), with a nonsignificant trend also observed between peak parasitemia and risk of developing ALT ≥ 2.5 × ULN (OR 3.72; 95% CI: 0.77–18.03; *P* = 0.10; Table 3). No relationship between PCt_1/2_ and ALT elevations was observed (Table 3).

**Table 3:** Association of explanatory parameters with elevated peak ALT in induced blood-stage malaria

Explanatory parameter	Peak ALT		Logistic regression		Logistic regression with fixed cohort effect	
	< 2.5 × ULN (*n* = 21)	≥ 2.5 × ULN (*n* = 11)	OR (95% CI)	*P*-value	OR (95% CI)	*P*-value
Median parasite clearance half-life (hours), (IQR)	4.70 (4.12–5.69)	4.36 (3.16–5.40)	0.69 (0.34–1.39)	0.30	1.06 (0.43–2.60)	0.90
Median peak parasitemia (parasites/mL), (IQR)	45,156 (12,922–78,227)	96,020 (52,370–201,433)	3.72 (0.77–18.03)	0.10	1.78 (0.17–18.72)	0.63
Median parasite clearance burden (IQR)	0.96 (0.71–1.41)	2.06 (1.15–2.32)	4.28 (1.26–14.59)	**0.020**	4.04 (0.38–49.84)	0.25
Median maximum temperature (°C), (IQR)	38.4 (37.3–39.1)	38.7 (38.4–39.3)	1.92 (0.78–4.69)	0.15	2.28 (0.65–7.97)	0.20
Median maximum clinical score (IQR)	4 (2–6)	6 (4–11)	1.19 (0.98–1.45)	0.085	1.09 (0.87–1.37)	0.45
Median maximum C-reactive protein × ULN (to EOS), (IQR)	5.4 (2.6–9.2)	12.0 (7.2–14.2)	1.43 (1.10–1.86)	**0.008**	1.45 (1.05–2.01)	**0.025**
Median lactate dehydrogenase × ULN (to EOS), (IQR)	0.96 (0.86–1.08)	1.25 (1.05–1.36)	1.56 (1.08–2.24)	**0.017**	1.43 (0.94–2.16)	0.093

ALT = alanine transaminase; EOS = end of study; IQR = interquartile range; OR = odds ratio; ULN = upper limit of normal. Odds ratio refer to a 1-unit change in the measure of the explanatory parameter (e.g., 1°C for maximum temperature). Statistically significant *P*-values < 0.05 are highlighted in bold.

The PCB (Table 3) was a significant risk factor for developing a peak ALT of ≥ 2.5 × ULN, with an OR of 4.28 (95% CI: 1.26–14.59; *P* = 0.020) for a one log_10_ increase in PCB. A significant correlation between log_10_ peak ALT and log_10_ PCB (*R* = 0.48; *P* = 0.005; Supplemental Figure 1) was also observed. In keeping with its more rapid antimalarial effect, the PCB was higher in the artefenomel-treated subjects than that in chloroquine-treated subjects (median 2.18 [IQR: 2.13–2.37] versus 0.95 [IQR: 0.71–1.22]; *P* < 0.001; Table 2).

**Table 2:** Summary of parasitemia-related outcomes in induced blood-stage malaria studies

Drug	Cohort	Median peak parasitemia (parasites/mL), (IQR)	Median parasite clearance half-life (hours), (IQR)	Median PCB* (IQR)
Artefenomel	*n* = 8	135,103 (69,107–189,959)	3.60 (3.11–4.31)	2.18 (2.13–2.37)
Chloroquine	C1 (*n* = 8)	10,367 (6,399–12,621)	5.48 (4.35–5.68)	0.63 (0.44–0.83)
Chloroquine	C2 (*n* = 8)	65,109 (25,839–130,410)	4.73 (4.23–5.42)	1.16 (0.83–1.39)
Chloroquine	C3 (*n* = 8)	74,243 (50,089–117,483)	5.59 (4.30–6.09)	1.18 (0.95–1.33)
Chloroquine overall	*n* = 24	41,766 (11,923–78,842)	5.28 (4.33–5.73)	0.95 (0.71–1.22)
Total	*n* = 32	57,554 (13,112–117,483)	4.40 (3.86–5.68)	1.16 (0.75–2.09)

IQR = interquartile range; PCB = parasite clearance burden.

* Parasite clearance burden was calculated by subtracting the parasitemia at 24 hours post-treatment initiation from the peak parasitemia on the log_10_ scale and is reported as a log-fold change.

#### Markers of inflammation and other laboratory parameters.

There was an association between CRP and the likelihood of ALT elevation: with each increase in the maximum CRP by 4 mg/L (ULN = 4 mg/L), the likelihood of a subject developing peak ALT ≥ 2 × ULN increased by 1.43-fold (95% CI: 1.10–1.86); *P* = 0.008, Table 3. Likewise, the peak CRP (× ULN) was associated with peak ALT (*R* = 0.44; *P* = 0.012; Supplemental Table 8).

#### Multivariable analysis.

On multivariable analysis, peak CRP remained a significant risk factor for developing an ALT ≥ 2.5 × ULN after controlling for the PCB but not after controlling for the cohort effect. After controlling for the PCB, artefenomel drug treatment no longer predicted the development of peak ALT ≥ 2.5 × ULN (OR 5.0 [95% CI: 0.91–27.47; *P* = 0.064] to 0.84 [95% CI: 0.05–15.19; *P* = 0.91]). No other pre-treatment laboratory parameter predicted the development of an ALT ≥ 2.5 × ULN after controlling for the cohort effect (Supplemental Table 1).

#### Treatment outcomes.

There was no association between maximum drug concentration (*C*_max_), time to maximum drug concentration (*T*_max_), or area under the parasitemia curve at 96 hours post-treatment, for artefenomel or chloroquine, and the timing or severity of ALT elevations.

#### Acetaminophen use and alternative medical diagnoses.

In the chloroquine study, the median peak ALT did not differ between cohort 2 (1.63 × ULN [IQR: 0.82–3.46]), where ibuprofen was administered for symptom relief, and cohort 3 (1.39 × ULN [IQR: 1.05–2.5]), where acetaminophen was the preferred treatment (Supplemental Figure 2). Review of the source documents did not reveal any alternative causes (including exercise) for the observed LFT abnormalities.

### Phase IIA study of artefenomel in Thailand.

The median (IQR; range) age of the 41 participants was 26 (21–30; 18–60) years, and 32 (78%) were male. The mean (range) parasite count for *P. vivax*–infected patients was 15,905/μL (5,010–53,400).^25^

**Figure 3.**
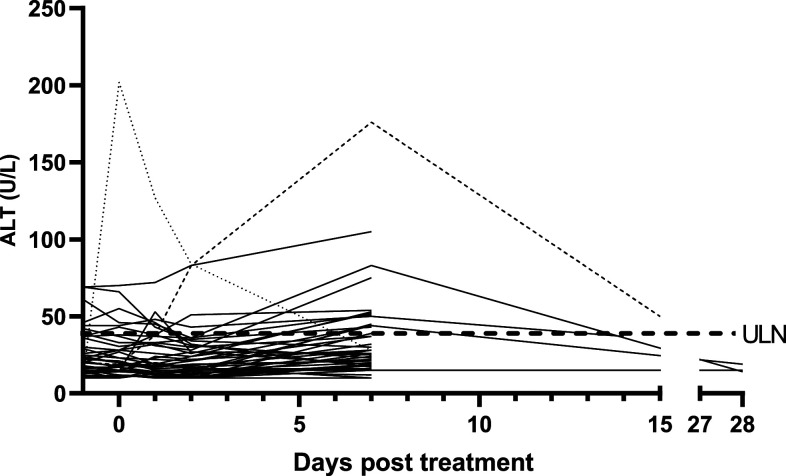
Phase IIA artefenomel study: course of ALT levels. Changes in ALT levels (U/L) over time for all 41 subjects. Day 1 represents the day 0 pre-treatment value and day 0 represents the first ALT value post-treatment. Data are displayed in this way to ease the graphical representation of the data. For ease of interpretation subjects, 2–19 and 1–22 are represented by dotted and dashed lines, respectively. The ULN for ALT in male and female subjects (40 IU/L) from the assay used in the study has been included for reference. ALT = alanine transaminase; ULN = upper limit of normal.

The median ALT increased from 21 IU/L pre-treatment to 30 IU/L at day 7 (*P* < 0.001). One patient had an ALT elevation of ≥ 2.5 × ULN, with a maximum absolute increase of 156 U/L (20 U/L on day 0, 176 U/L on day 7, and improving to 50 U/L on day 15). This patient was treated with a 200 mg dose of artefenomel, the lowest dose used in the study (Figure 3).

### Sabah Malaysia.

Eighty-five patients had day 0 and day 7 ALT measures available and were included in the analysis. The median (IQR; range) age was 16 (11–64; 3–65) years, and 63 (74%) were male. The median (IQR) parasite count at day 0 was 4,048/μL (1,523–8,120).

**Figure 4.**
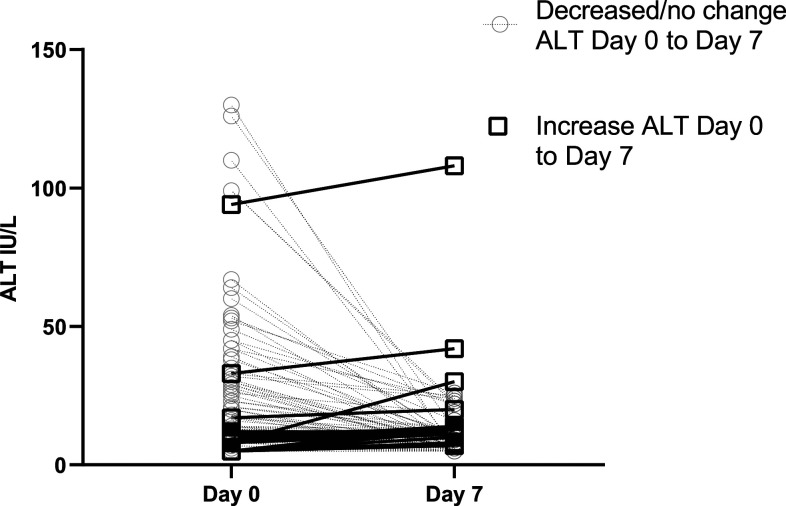
Malaysia studies: course of ALT levels. Day 0 (pre-treatment) and day 7 ALT results for all subjects segregated into those who had an increase in ALT and those who did not. ALT= alanine transaminase.

Forty-four (51.8%) of the 85 subjects were treated with artesunate/mefloquine, 32 (37.7%) with chloroquine, 7 (8.2%) with artemether/lumefantrine and primaquine, and 2 (2.4%) with chloroquine and primaquine. Among those treated with chloroquine, the median ALT decreased from 14 (IQR: 7–29) at day 0 to 7.5 (IQR: 5–11) at day 7 (*P* < 0.001), whereas in those treated with artemether/lumefantrine, the median ALT decreased from 17 (IQR: 11–35) at day 0 to 10 (IQR: 7–13) at day 7 (*P* < 0.001). No patient developed an ALT ≥ 2.5 × ULN (Figure 4).

## DISCUSSION

Asymptomatic transaminase elevations ≥ 2.5 × ULN without concomitant increases in bilirubin occurred in 11/32 (34%) subjects in the two *P. vivax* IBSM studies. Abnormalities generally began 3 days post-treatment, peaking 5–8 days post-treatment, with resolution before the EOS. Peak ALT was typically greater than peak AST. We were unable to identify a single factor that accounted for all the observed transaminase elevations in the *P. vivax* IBSM studies. Moreover, we did not identify any pre-treatment parameter that predicted subsequent development of transaminase elevations. However, we observed statistically significant associations between transaminase elevations and PCB and CRP.

Transaminase elevations mirroring those seen in the *P. vivax* IBSM studies were rare in the clinical *P. vivax* studies, occurring in only 1/41 (2.4%) Thai patients treated with artefenomel and in none of the 76 Malaysian patients treated with chloroquine or artemisinin combination therapy. Consistent with these findings, in a previous phase II study of artefenomel (800 mg) in combination with piperaquine (640 mg, 960 mg, or 1,440 mg) for treatment of *P. falciparum* malaria, only 1/448 (0.2%) subjects experienced a grade 3 (5.1–10 × ULN) transaminase elevation.^26^ Transaminase elevations were also not observed in a phase I study of artefenomel in 26 healthy volunteers without malaria.^23^

In the VISs, although the risk of developing elevations of ALT ≥ 2.5 × ULN was higher among subjects treated with artefenomel than that among those treated with chloroquine, this association disappeared when corrected for other factors, notably the PCB (Supplemental Table 7). Of significance, artefenomel has a more rapid parasiticidal effect than chloroquine.^25,26,31^

The PCB is a composite of the rate of parasite clearance (i.e., PCt_1/2_) and peak parasitemia around the time of treatment, and thus, it represents the biomass of parasites cleared immediately post-treatment. As such, it distinguishes between artefenomel (a fast-killing drug) and chloroquine (a drug with a slower effect on parasite clearance), while still accounting for the overall reduction in parasite biomass post–antimalarial treatment (Table 2). Peak parasitemia has previously been identified as a risk factor for LFT abnormalities in malaria VISs,^39^ but in our analysis, it had a weaker relationship with peak ALT than PCB.

We have previously reported post-treatment transaminase elevations of > 5 × ULN in 4/6 (66%) subjects in a *P. vivax* IBSM study where a different parasite isolate from a different donor was used. In that study, treatment was also with the fast-acting drug artemether/lumefantrine,^2^ and the high proportion of subjects with LFT abnormalities, in the context of rapid parasite clearance and likely high PCB, is consistent with findings reported here. LFT abnormalities similar to those described in the *P. vivax* IBSM studies have been observed in IBSM studies with *P. falciparum* 3D7^16^ as well as in *P. falciparum* studies where infection was induced by sporozoites.^7,15^ Therefore, the observed LFT abnormalities are unlikely to be due to a specific effect from the species or strain used during these studies nor the route of infection.

In clinical trials of antimalarials, clinically significant transaminase elevations occurring after treatment have not been commonly reported. Assessments of liver function during the day 5–8 window where ALT changes were observed in the IBSM studies may not have been universally undertaken.^24,40–46^ However, even when testing has occurred 5–8 days post-treatment, transaminase elevations mirroring those observed in *P. vivax* IBSM studies appear to be uncommon in *P. falciparum*–infected patients treated with artemisinin combination therapies (0.2–7.8%).^19,47–53^ For example, in a recent randomized clinical trial in West Africa where four different artemisinin combination therapies were evaluated in 4,710 patients experiencing 8,640 malaria episodes (7,119 *P. falciparum*, 146 *P. malariae*, 31 *Plasmodium ovale*, and 17 mixed infections), the LFT was undertaken on days 0 (pre-dose), 3, 7, and 28. In this study, LFT changes resembling those we report here were rare, being reported after treatment with any of the four drug combinations in 0.9–3.9% of the 8,640 treatment encounters, and were more commonly seen in children < 5 years of age.^19^ All patients were treated with artemisinin-based regimens characterized by rapid parasite clearance, and thus more likely to have a high PCB.

One possible explanation for the lower frequency of post-treatment increases in ALT in malaria-endemic settings is that there may be a reduced susceptibility to hepatic injury due to previous exposure to malaria,^54^ with resulting modulation of the host immune response.^55^ This may also explain why ALT increases post-treatment occur more often in returned travelers,^5,47^ in whom most malaria episodes will be a primary infection. Alternatively, it is also possible that genetic polymorphisms or other host characteristics such as iron deficiency could influence susceptibility to transaminase elevations following malaria treatment.^56,57^

Higher maximum CRP was associated with an increased risk of a peak ALT of ≥ 2.5 × ULN, suggesting that systemic inflammation likely contributes to the development of post-treatment LFT abnormalities in VISs, as previously postulated.^39^ Rodent malaria studies have suggested that TNF-α generated during malaria infection and free heme, produced following rapid hemolysis, act together to induce hepatocyte apoptosis and transaminase leakage.^58–61^ Testing for additional markers of hemolysis, free heme, and TNFα would also be worth undertaking in future studies. In addition to the effects of systemic inflammation and hemolysis, it is possible, though speculative, that liver injury in primary infections may also result from killing of *P. vivax* parasites potentially sequestered in the liver, as now recognized to occur in the spleen,^62^ possibly exacerbating localized hepatic inflammation.

The relatively small number of subjects in this study limits statistical power, and a larger dataset would be required for a more robust multivariable analysis. Differences between cohorts meant comparisons across cohorts was difficult and could confound the analysis. In the case of PCB, it is strongly affected by both the day of treatment and the antimalarial used. In addition, as it is calculated solely from two data points, the measure may be susceptible to bias and should be prospectively evaluated in future studies.

Post-treatment LFT changes appear to be more common in *P. vivax* IBSM than natural infection. Although treating physicians should remain vigilant, these changes have to date been asymptomatic, self-limiting, and not associated with adverse clinical outcomes. Researchers and pharmaceutical companies should become familiar with these findings so that the mechanisms underlying this phenomenon can be better understood and alternate explanations of the causation of LFT changes can be considered in the development of antimalarials. LFT on days 3, 5, and 7 post-treatment initiation should be incorporated, where possible, into the study design of antimalarial clinical trials.

## Supplemental materials, table and figures

Supplemental materials
