# Supplementary material for: Liver Function Test Abnormalities in Experimental and Clinical Plasmodium vivax Infection
Source: Am J Trop Med Hyg. 2020 Aug 17;103(5):1910–7. doi: 10.4269/ajtmh.20-0491 (PMC7646782; doi:10.4269/ajtmh.20-0491)
Supplement: Supplementary file 1 [file tpmd200491.SD1.pdf]

Supplemental Material

The following are supplemental materials and will be published online only

Liver function test abnormalities in induced blood stage *Plasmodium vivax* malaria: supplementary material

Malaria clinical score

The malaria clinical score comprises 14 variables (see below), each being graded between 0 and 3 based on the severity of impairment of the subjects' activity (0 = absent; 1 = mild, no impairment of usual activity; 2 = moderate, no impairment of activities of daily living; 3 = severe, activities of daily living impaired). The scores for all 14 variables are then added together to give the malaria clinical score.

- Headache
- Myalgia (muscle ache)
- Arthralgia (joint ache)
- Fatigue/lethargy
- Malaise (general discomfort/uneasiness)
- Chills/Shivering/Rigors
- Sweating/hot spells
- Anorexia
- Nausea
- Vomiting
- Abdominal discomfort
- Fever
- Tachycardia
- Hypotension

Supplemental Figure 1. Peak ALT vs parasite clearance burden in IBSM.

ALT (alanine transaminase), ULN (upper limit of normal). Relationship between peak ALT (log_{10}) and parasite clearance burden (log_{10}) (Pearson's correlation).

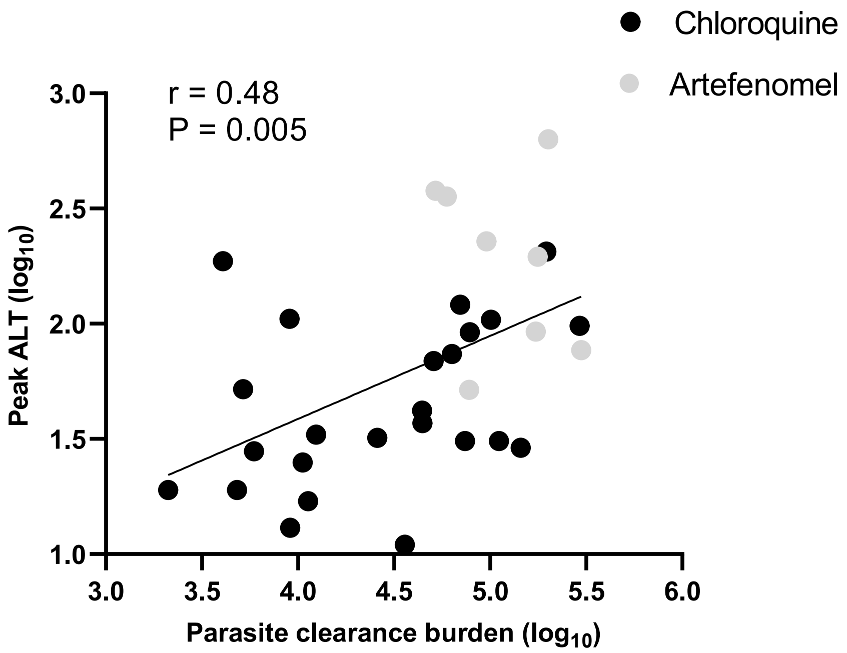
Chloroquine
Artefenomel
r = 0.48
P = 0.005
Peak ALT (log10)
3.0
2.5
2.0
1.5
1.0
3.0
3.5
4.0
4.5
5.0
5.5
6.0
Parasite clearance burden (log10)

Supplemental Figure 2. Peak ALT Chloroquine Cohort C2 (no acetaminophen) vs Chloroquine Cohort C3 (with acetaminophen) in IBSM study subjects.

ALT (alanine transaminase), ULN (upper limit of normal). Peak ALT of subjects in Chloroquine cohort C2 (subjects did not receive acetaminophen) compared to subjects in Chloroquine cohort C3 (subjects received acetaminophen). Median and IQR peak ALT values for subjects in Chloroquine cohort 2 and Chloroquine cohort 3 are demonstrated.

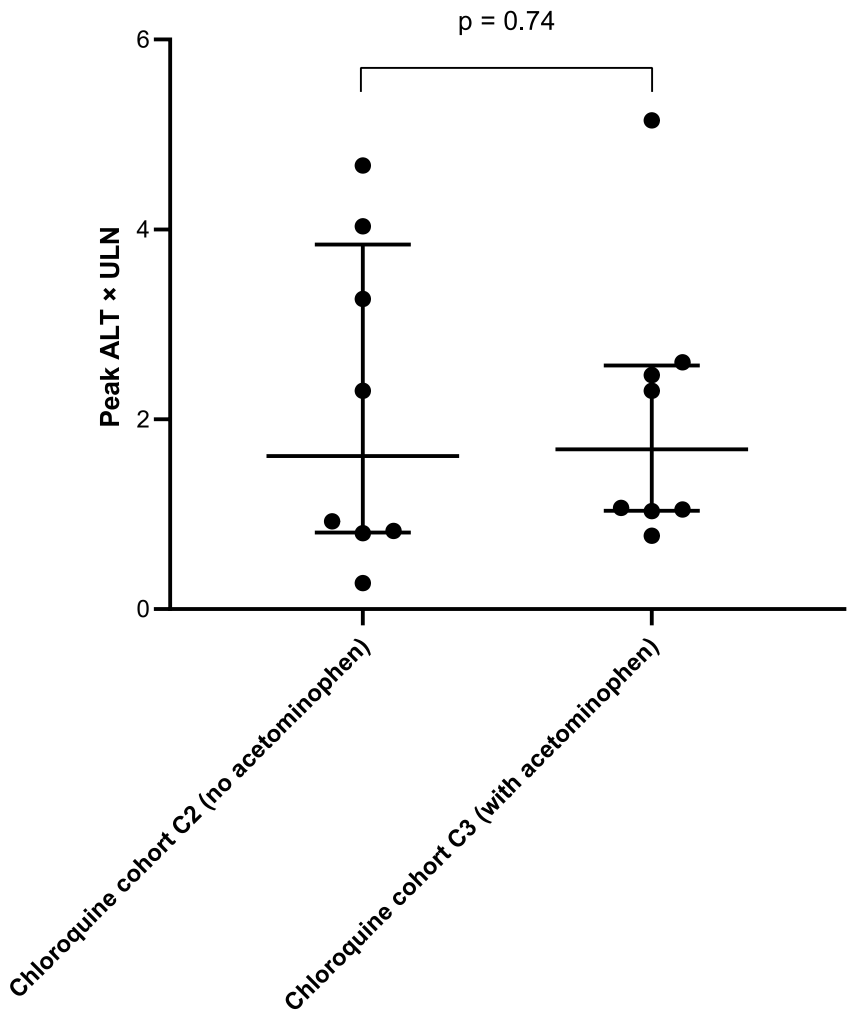
p = 0.74
Peak ALT × ULN
0
2
4
6
Chloroquine cohort C2 (no acetominophen)
Chloroquine cohort C3 (with acetominophen)

Supplemental Table 1. Association of laboratory parameters with elevated peak ALT in IBSM.

Explanatory Variable	Peak ALT		Logistic regression		Logistic regression with fixed cohort effect	
	$<$ 2.5 × ULN (n = 21)	$\geq$ 2.5 × ULN (n = 11)	OR (95% CI)	p	OR (95% CI)	p
**Median peak parasitemia Pre-treatment (log_{10} parasites/mL), (IQR)**	4.39 (3.90 - 4.79)	4.88 (4.48 - 5.07)	4.37 (0.90 - 21.35)	0.068	2.97 (0.29 - 30.13)	0.36
**Median nadir Neutrophils × LLN (To EOS), (IQR)**	1.14 (1.07 - 1.34)	0.94 (0.81 - 1.3)	0.10 (0.006 - 1.68)	0.11	0.10 (0.004 - 2.24)	0.15
**Median nadir neutrophils × LLN (Pre-treatment), (IQR)**	2.09 (1.65 - 2.27)	1.64 (1.3 - 1.88)	0.26 (0.06 - 1.23)	0.090	0.17 (0.023 - 1.23)	0.080
**Median platelets × LLN (To EOS) , (IQR)**	0.97 (0.84 - 1.07)	0.83 (0.73 - 0.85)	0.001 (<0.001 - 0.79)	**0.042**	<0.001 (<0.001 - 0.64)	**0.039**
**Median nadir platelets × LLN (Pre-treatment), (IQR)**	1.17 (0.99 - 1.36)	1.01 (0.85 - 1.24)	0.04 (<0.001 - 1.47)	0.079	0.008 (<0.001 - 2.22)	0.093
**Median nadir lymphocytes × LLN (To EOS), (IQR)**	0.85 (0.61 - 1.04)	0.64 (0.54 - 0.73)	0.01 (<0.001 - 0.98)	**0.049**	0.04 (<0.001 - 9.08)	0.24
**Median nadir lymphocytes × LLN (Pre-treatment), (IQR)**	0.94 (0.61 - 1.51)	0.64 (0.54 - 0.73)	0.06 (0.003 - 1.14)	0.061	0.01 (<0.001 - 3.13)	0.12
**Median nadir white cell count × LLN (To EOS), (IQR)**	1.03 (0.97 - 1.2)	0.89 (0.74 - 1.17)	0.06 (0.002 - 2.26)	0.13	0.003 (<0.001 - 0.68)	**0.035**
**Median nadir white cell count × LLN (Pre-treatment), (IQR)**	1.31 (1.11 - 1.63)	0.97 (0.86 - 1.37)	0.60 (0.005 - 0.84)	**0.036**	0.02 (<0.001 - 1.00)	0.050
**Median CRP × ULN (Pre-treatment), (IQR)**	n = 11 0.60 (0.2 - 1.6)	n = 5 2.6 (0.8 - 4.8)	1.38 (0.86 - 2.22)	0.18		
**Median LDH × ULN (Pre-treatment), (IQR)**	0.69 (0.60 - 0.78)	0.79 (0.66 - 0.87)	1.41 (0.83 - 2.37)	0.20	1.04 (0.56 - 1.96)	0.90
**Median chloroquine AUC_{96} (µg/mL × h), (IQR)**	n = 18 59.25 (50.31 - 69.24)	n = 6 58.24 (50.32 - 67.10)	0.98 (0.92 - 1.05)	0.61	1.00 (0.92 - 1.09)	0.93
**Median chloroquine C_{max} (µg/mL), (IQR)**	1.03 (0.88 - 1.23)	0.93 (0.75 - 1.25)	0.29 (0.009 - 10.01)	0.50	0.89 (0.01 - 65.69)	0.96
**Median artefenomel AUC_{96} (µg/mL × h), (IQR)**	n = 3 4.01 (2.45 - 4.87)	n = 5 3.72 (3.43 - 4.44)	1.30 (0.34 - 4.95)	0.71		
**Median artefenomel C_{max} (µg/mL), (IQR)**	0.66 (0.32 - 0.75)	0.54 (0.48 - 0.69)	2.57 (0.002 - 3589)	0.80		

ALT (alanine transaminase), CRP (C-reactive Protein), LDH (lactate dehydrogenase), C_{max} (maximum concentration of drug), AUC_{96} (area under the curve for drug dose 96 hours after drug administration), ULN (upper limit of normal), OR (odds ratio), EOS (end of study). OR refer to a 1-unit change in the measure of the explanatory parameter (e.g. 1°C for maximum temperature).

Significant effects and associations ($p < 0.05$) are highlighted in bold. Continuous measures are described with median (IQR). Results from simple binary logistic regression modelling the probability of elevated peak ALT (ALT $\geq$ 2.5 ×ULN) are presented (logistic regression) and also adjusted for cohort by including a fixed effect for cohort (logistic regression with fixed cohort effect).

Supplemental Table 2. Inflammatory parameters per cohort in IBSM.

Cohort	Statistical measure	^Maximum temperature (°C)	*Maximum Clinical score	Peak CRP × ULN (U/L)	Peak CRP Relative to baseline (U/L)
**Artefenomel (n = 8)**	Median (IQR)<br>n (%) raised^*	39.2 (38.7 - 39.7)<br>8 (100%)	8.5 (6.0 - 12.0)<br>7 (87.5%)	11.6 (8.9 - 13.8)	58 (44.5 - 69.0)
**Chloroquine C1 (n = 8)**	Median (IQR)<br>n (%) raised^*	37.3 (36.8 - 38.1)<br>3 (37.5%)	3.5 (0.5 - 4.0)<br>0 (0%)	1.9 (0.9 - 4.3)	6.5 (3.5 - 14.0)
**Chloroquine C2 (n = 8)**	Median (IQR)<br>n (%) raised^*	38.6 (38.3 - 38.9)<br>7 (87.5%)	5.0 (2.0 - 10.5)<br>4 (50.0%)	9.9 (5.2 - 12.1)	35.5 (26.0 - 51.0)
**Chloroquine C3 (n = 8)**	Median (IQR)<br>n (%) raised^*	38.7 (38.5 - 39.6)<br>7 (87.5%)	6.0 (4.0 - 6.5)<br>5 (62.5%)	7.8 (5.5 - 9.3)	32.5 (24.5 - 46.5)
**Overall (n = 32)**	Median (IQR)<br>n (%) raised^*	38.6 (38.1 - 39.2)<br>25 (78.1%)	5.5 (2.5 - 8.0)<br>16 (50.0%)	7.5 (4.8 - 11.6)	35.8 (14.5 - 54.0)

CRP (C-reactive Protein), ULN (upper limit of normal), IQR (interquartile range) ^Raised maximum temperature ≥ 37.5°C, *raised maximum Clinical Score ≥ 6. Maximum values for temperature and clinical score within 3 days either side of treatment.

Supplemental Table 3. Pharmacokinetic parameters per cohort in IBSM.

Identifier		Antimalarial	
**Cohort**	**Statistical measure**	**C_{max} (µg/mL)**	**AUC_{96} (µg/mL × h)**
**Artefenomel**	Median (IQR)	0.60 (0.43 - 0.72)	3.86 (3.08 - 4.66)
**Chloroquine C1**	Median (IQR)	1.24 (1.11 - 1.46)	66.28 (59.56 - 79.36)
**Chloroquine C2**	Median (IQR)	0.88 (0.73 - 1.02)	52.88 (41.48 - 60.37)
**Chloroquine C3**	Median (IQR)	0.99 (0.82 - 1.09)	58.24 (48.24 - 64.48)

C_{max} (maximum concentration of drug), AUC_{96} (area under the curve for drug dose 96 hours after drug administration), IQR (interquartile range).

Supplemental Table 4. Pain relief medication per cohort in IBSM.

Identifier		Pain relief medication	
**Cohort**	**Statistical measure**	**Total acetaminophen (g)**	**Total ibuprofen (g)**
**Artefenomel**	Median (IQR)<br>n (%) positive^	3.0 (2.5 - 5.5)<br>8 (100%)	2.0 (1.8 - 3.2)<br>8 (100%)
**Chloroquine C1**	Median (IQR)<br>n (%) positive^	-<br>-	1.0 (0 - 2.0)<br>5 (62.5%)
**Chloroquine C2**	Median (IQR)<br>n (%) positive^	-<br>-	2.4 (1.5 - 4.6)<br>8 (100%)
**Chloroquine C3**	median (IQR)<br>n (%) positive^	8.8 (5.0 - 10.0)<br>8 (100%)	-<br>-

IQR (interquartile range). ^Subject who received pain relief.

Supplemental Table 5. Results of backwards stepwise logistic regression for the risk of elevated peak ALT in IBSM with $PCT_{1/2}$ included in all steps of the models.

Model	Measure	OR (95% CI)	p
**Including cohort effect**	**$PCT_{1/2}$**	1.81 (0.57 - 5.69)	0.31
	**Nadir Platelets × LLN^**	2.79 (1.12 - 6.93)	0.027
	**Cohort - ref Artefenomel**	1	
	**Chloroquine C1**	0.16 (0.006 - 3.89)	0.26
	**Chloroquine C2**	0.04 (0.001 - 1.36)	0.075
	**Chloroquine C3**	0.03 (< 0.001 - 1.15)	0.059
**No cohort effect**	**$PCT_{1/2}$**	0.95 (0.39 - 2.33)	0.92
	**Peak CRP (relative to baseline)**	1.06 (1.01 - 1.12)	0.013

CRP (C-reactive Protein), LLN (lower limit of normal), $PCT_{1/2}$ (parasite clearance half-life) OR (odds ratio). ^Effect size (OR and 95% CI) are converted to per 0.1 unit decrease in × LLN. The order of variables removed in the model including cohort effect were were burden, white cell count, and then Peak CRP relative to baseline. The order of variables removed in the model did not include cohort effect were platelets, burden, and then white cell count.

Supplemental Table 6. Results of backwards stepwise logistic regression for the risk of elevated peak ALT in IBSM with parasite clearance burden included in all steps of the models.

Model	Measure	OR (95% CI)	p
**Including cohort effect**	**PCB (log_{10} scale)**	3.60 (0.19 - 68.81)	0.40
	**Peak CRP (relative to baseline)**	1.06 (1.00 - 1.13)	0.044
	**Cohort - ref Artefenomel**	1	
	**Chloroquine C1**	8.63 (0.026 - 2889)	0.47
	**Chloroquine C2**	2.89 (0.05 - 153)	0.60
	**Chloroquine C3**	1.77 (0.03 - 114)	0.79
**No cohort effect**	**PCB (log_{10} scale)**	1.86 (0.42 - 8.17)	0.41
	**Peak CRP (relative to baseline)**	1.05 (1.00 - 1.11)	0.054

CRP (C-reactive Protein), OR (odds ratio), PCB (parasite clearance burden). ^Effect size (OR and 95% CI) are converted to per 0.1 unit decrease in × LLN. The order of variables removed in the model including cohort effect were $PCT_{1/2}$, platelets and then white cell count. The order of variables removed in the model did not include cohort effect was white cell count, $PCT_{1/2}$ and then platelets.

Supplemental Table 7. Logistic regression for the risk of elevated peak ALT in IBSM with antimalarial drug, drug and treatment day and drug and parasite clearance burden.

Model	Measure	OR (95% CI)	p
**Drug (n = 32)**	**Drug**		
	Chloroquine	Reference	
	Artefenomel	5.00 (0.91 - 27.47)	0.064
**Drug and Treatment day (n = 31)**	**Drug**		
	Chloroquine	Reference	
	Artefenomel	4.58 (0.73 - 28.65)	0.10
	**Treatment Day**		
	Treatment day 8	Reference	
	Treatment day 10	2.55 (0.23 - 27.71)	0.44
**Drug and PCB (n = 32)**	**Drug type**		
	Chloroquine	Reference	
	Artefenomel	0.84 (0.05 - 15.19)	0.91
	PCB (log_{10} scale)	4.71 (0.61 - 36.41)	0.14

OR (odds ratio), PCB (parasite clearance burden). Risk of ALT ≥ 2.5 × ULN depending on drug, drug and treatment day, and drug and parasite clearance burden.

Supplemental Table 8. Pearson correlation of with peak ALT in IBSM.

Clinical measure	Correlation	p
**Peak AST**	**0.99**	**< 0.001**
**Peak Bilirubin**	-0.10	0.57
**Peak LDH**	**0.58**	**< 0.001**
**$PCT_{1/2}$**	-0.26	0.16
**Pre-treatment Peak parasitemia**	**0.36**	**0.044**
**Overall Peak parasitemia**	**0.39**	**0.027**
**PCB**	**0.58**	**< 0.001**
**Nadir Neutrophils**	-0.09	0.62
**Nadir Platelets**	-0.26	0.15
**Nadir Lymphocytes**	**-0.41**	**0.018**
**Nadir White cell count**	-0.03	0.87
**Peak Clinical Score**	**0.42**	**0.017**
**Maximum Temperature**	0.29	0.11
**Peak CRP**	**0.44**	**0.012**

ALT (alanine transaminase), AST (aspartate transaminase, LDH (lactate dehydrogenase), CRP (C-reactive Protein), LDH (lactate dehydrogenase), $PCT_{1/2}$ (parasite clearance half-life), PCB (parasite clearance burden). LFT and haematology results are in units of overall peak/minimum times ULN or LLN. effects and associations ($p < 0.05$) are highlighted in bold.
